# MULTIOMIC SYSTEMS ANALYSIS OF LIFESPAN-EXTENDING INTERVENTIONS IN MOUSE TISSUES

**DOI:** 10.1093/geroni/igad104.3000

**Published:** 2023-12-21

**Authors:** Kengo Watanabe, Tomasz Wilmanski, Priyanka Baloni, Max Robinson, Oliver Fiehn, Robert Moritz, Richard Miller, Noa Rappaport

**Affiliations:** Institute for Systems Biology, Seattle, Washington, United States; Institute for Systems Biology, Seattle, Washington, United States; Purdue University, West Lafayette, Indiana, United States; Institute for Systems Biology, Seattle, Washington, United States; UC Davis, Davis, California, United States; Institute for Systems Biology, Seattle, Washington, United States; University of Michigan, Ann Arbor, Michigan, United States; Institute for Systems Biology, Seattle, Washington, United States

## Abstract

Aging is associated with dysregulation of molecular, cellular, and physiological processes. Nutritional and pharmacological interventions with different postulated modes of action have been shown to extend lifespan and delay aging-related diseases in model organisms, suggesting the existence of core cellular processes that mediate the effect on lifespan and healthspan. However, these underlying molecular processes remain largely uncharacterized. In this study, we analyzed multiomic tissue-derived data of lifespan-extending interventions in mice including metabolomics and proteomics from the NIA Longevity Consortium and previously reported transcriptomics. We applied three systems techniques, differential rank conservation (DIRAC) analysis, weighted gene co-expression network analysis (WGCNA), and mouse genome-scale metabolic model (GEM) reconstruction, to the mouse liver proteomic and transcriptomic datasets of lifespan-extending interventions (e.g., acarbose, 17α-estradiol, and rapamycin). We found that these interventions generally strengthen the rank conservation of biological processes and shift metabolic fluxes, with fatty acid metabolism emerging as a common process affected by multiple interventions. We then applied these approaches to an expanded experimental data with additional interventions (e.g., canagliflozin) and tissues (e.g., kidney, muscle, and plasma), confirming our results and further observing varied inter-omic and inter-tissue patterns exerted by lifespan-extending interventions. Our findings highlight the potential of integrative systems analysis to elucidate common and unique cellular and physiological changes relating to aging and lifespan-extending interventions, which have translational potential as preventive and prognostic measures to improve human health.

